# Influence of the Rater’s Gender on Assessing Facial Beauty in Adult Patients With Vertical and Horizontal Growth Patterns

**DOI:** 10.7759/cureus.63142

**Published:** 2024-06-25

**Authors:** Marwa Ali Albitar, Ahmad S Burhan, Mohammad Y Hajeer, Fehmieh R Nawaya, Wagd Khlaid Roumieh

**Affiliations:** 1 Department of Orthodontics, Faculty of Dentistry, University of Damascus, Damascus, SYR; 2 Department of Pediatric Dentistry, Faculty of Dentistry, Syrian Private University, Damascus, SYR

**Keywords:** facial aesthetic evaluation, facial esthetics, lateral photographs, frontal photographs, horizontal growth pattern, vertical growth pattern, raters, panel of assessors, attractiveness

## Abstract

Background

The evaluation of attractiveness varies from one civilization, culture, and environment to another and between individuals. Gender can also play a role in determining the standards of attractiveness. The purpose of this study was to evaluate the effect of the rater’s gender on the assessment of adult facial attractiveness with a vertical and horizontal growth pattern in patients with skeletal Class I malocclusion.

Methodology

The study sample comprised extraoral photos taken before the treatment of 120 patients (30 males and 30 females in each group) with skeletal Class I malocclusion and vertical and horizontal growth patterns according to the Bjork sum aged between 18 and 25 years. A panel of 30 laypersons (aged 19-25 years with an average age of 23 ± 0.53 years), including raters from both genders, were selected equally using a disproportionate stratified sampling method through a computer-generated list. The raters used the visual analog scale (VAS) to provide a score for each photograph’s aesthetic quality. The most attractive group, which received the greatest aesthetic score, and the least attractive group, which received the lowest aesthetic score, were the two groups formed based on each photograph’s mean aesthetic scores. Overall, 13 patients were chosen for each group. Subsequently, the average assessment score for every patient photo set was determined. Independent-sample t-tests were employed to ascertain if the raters’ gender made a statistically significant difference in assessing patients with vertical and horizontal growth patterns.

Results

There were statistically significant differences between the gender of raters in evaluating female patients with vertical growth patterns (p < 0.001), where the average rating of the female raters was significantly greater than that of the male raters in evaluating female patients. In addition, there were statistically significant differences between the gender of raters in evaluating female patients with horizontal growth patterns (p = 0.009), where the average rating of the male raters was significantly greater than that of the female raters in evaluating female patients.

Conclusions

There is a limited effect of the rater’s gender in evaluating facial aesthetics. However, the facial features of female patients with long faces are preferred by females more than males, and males are more critical in evaluating these patients. On the other hand, males favor the facial features of female patients with short faces more than females, and females are more critical in evaluating these patients. These results suggest considering patients’ personal characteristics with vertical and horizontal growth patterns during diagnosis and treatment planning.

## Introduction

The concept of beauty does not depend on fixed principles, but rather its standards conflict between people and ethnic groups and according to social and economic customs and traditions [1]. Every person is considered an expert in evaluating facial beauty [2]. Over the years, as there have been significant changes in standards of facial beauty, orthodontists must be aware of what the population considers the ideal face for that time [3]. However, from a specialist’s point of view, successful orthodontic treatment does not always improve the patient’s facial beauty. Therefore, the result of treatment may be considered unsatisfactory from the patient’s point of view [4].

The dental aesthetic standard differs from the facial aesthetic standard, and the dental standard often does not include an aesthetic evaluation of the entire face. The following three elements should be considered when developing a system for evaluating facial beauty: how patients are presented to the evaluators, the characteristics of the evaluators, and the measurement method [5]. The literature includes studies that have evaluated facial beauty by presenting photographs, sketches, or shadows of the soft tissue profile to a panel of judges for evaluation [6-9].

There are various methods to evaluate facial beauty, each with advantages and disadvantages. One of the advantages of using soft tissue shadows in standard vertical-lateral radiographs is that it eliminates the impact of confounding factors when assessing aesthetics [10]. However, this method does not represent the entire face, and the smile cannot be evaluated using this method [11]. Frontal photographs are typically considered more attractive than side photographs [12], and it would be appropriate to display both images simultaneously to achieve a more accurate assessment of facial beauty [13].

In almost all previous studies, a rating panel was used to measure facial beauty, and as the concept of facial beauty may be related to geographic region, professional background, age, or gender of the rater, much attention was paid to comparing the different ratings of the rater panel [5]. However, research findings in this area have been contradictory, and differences in study design may be largely responsible for these conflicting results. In addition, factors related to the characteristics of the committee, such as professional background, age, and geographic region, may affect the evaluation results [14].

The gender of the person evaluating facial beauty affects the evaluation score [15]. Baker et al. found that females pay more attention to the eyes, while males pay more attention to the mouth. In addition, males pay more attention to dental beauty, regardless of the aesthetic background of the face, and this was found to be the opposite of what was found in females [16]. On the other hand, when additional research was conducted in other studies, there were no differences in aesthetic evaluation between resident genders [14,17-22].

After reviewing previous studies, it became clear that the potential influence of the evaluator’s gender on facial aesthetic evaluation remains controversial. There were also few studies evaluating patients with a vertical and horizontal growth pattern in skeletal Class I malocclusion. Therefore, ​​this study aimed to clarify how the gender of the evaluator can affect the evaluation of facial aesthetics in these patients.

## Materials and methods

Study design and settings

This was a cross-sectional study for descriptive and analytical aims. Patients’ photos served as the basis for data collection. Between February 2022 and October 2023, this study was conducted in the Department of Orthodontics, Faculty of Dentistry, University of Damascus (Damascus, Syria). The Local Research Ethics Committee of the Faculty of Dentistry granted ethical approval (reference number: UDDS-999-24022022/SRC-1450). The University of Damascus funded this project (reference number: 501100020595).

The evaluation panel and raters’ recruitment

The inclusion criteria for evaluators were undergraduate university students between the ages of 19 and 25 years, whose interests had no clear influence on their evaluation of beauty, such as painting and sculpture, and who were unfamiliar with orthodontics and patients. An invitation was sent to collect a sample of evaluators, and the research aims and evaluation methods were distributed to the students of the Faculty of Engineering at the University of Damascus. Subsequently, 67 students agreed to participate in the evaluation. Overall, 30 students were chosen using a disproportionate stratified sampling method through a computer-generated list to ensure equal distribution of males and females (i.e., 15 males and 15 females).

Sample collection and patient recruitment

Following an evaluation of 187 patients attending the Department of Orthodontics at the Faculty of Dentistry at the University of Damascus, 162 patients satisfied the inclusion criteria. An information sheet explaining the purpose and methodology of the study was given to each patient. Overall, 154 patients consented to participate (81 patients with a vertical growth pattern and 73 patients with a horizontal growth pattern), and informed consent was obtained from them. Subsequently, 120 of the 154 patients were randomly selected from the sample using a computer-generated sampling method to guarantee an equal distribution of males and females (i.e., 30 males and 30 females in each group). The inclusion criteria for patients were age between 18 and 25, skeletal Class I malocclusion, and vertical or horizontal growth pattern with Bjork sum 385-389 or 402-406. Exclusion criteria were prior orthodontic or prosthodontic treatment, a history of previous tooth extraction (excluding third molars), previous esthetic surgery in the facial area, craniofacial syndrome, and severe skeletal vertical or horizontal growth pattern.

Photographing method

**Figure 1:**
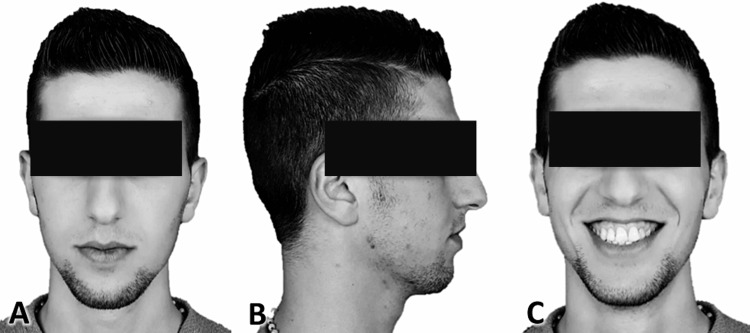
Photographic assessment of the patient’s face. A: Frontal relaxed facial expression, B: Profile relaxed facial expression, C: Frontal smiling expression.

A total of 120 patients were photographed in three positions using a Nikon D80 camera (Nikon D80; Nikon Corporation, Tokyo, Japan), 10.2 megapixels, and a 70-200 mm macro lens. The three positions included frontal relaxed, frontal during a smile, and relaxed profile [5]. Patients were asked to stand straight with their arms positioned freely on both sides of their body [19], taking into consideration the natural head position [20]. White backgrounds were used for the photos to prevent the surrounding colors from influencing the aesthetic assessment of the photos. The camera was positioned 150 cm away from the patient on a mount. To remove variables that could influence the evaluation of facial aesthetics, such as skin, eye, and hair color, all patient photos were turned into black-and-white using the Photos software (version 2018.18011.15918.0; Photos, Microsoft Corp., Seattle, WA, USA) (Figure 1). One researcher took all of the photos (MAA).

Evaluation of patients’ photos

Each patient’s photo set was printed on paper using a Ricoh 7500 printer in A6 size (105 × 148 mm) and then shown to the panel members in a silent, comfortable room. Each image was evaluated for a period not exceeding five seconds, with a 15-second interval after every 21 images. The panelists were asked to grade the facial appearance on a rating sheet from 0 to 100 mm on the visual analog scale (VAS) [18], with 0 as the least attractive and 100 as the most attractive. They were told that a score of 50 represented the average degree of facial aesthetics. In total, 120 patients were rated based on the means of the evaluation scores that were computed for each patient’s photo set. It was postulated that some persons should be placed in the middle between the two groups to distinguish between those who were expected to have scored highly on attractiveness assessments and those who were expected to score poorly (i.e., the least attractive persons). Consequently, the 13 most and 13 least attractive photographs for each gender were chosen, and four of those who scored averagely on the assessments were eliminated.

Statistical analysis

All statistical analysis was conducted using SPSS version 27 (IBM Corp., Armonk, NY, USA) software program. The Shapiro-Wilk test was used to check whether the data distribution was normal. Data were normally distributed; thus, the parametric tests of continuous quantitative variables were applied to analyze the data of this study. The mean values and the standard deviation for the facial beauty of participants were calculated. An independent-sample t-test was used to study the significance of the differences in the average scores of facial beauty between the sexes of the raters at the significance level (p = 0.05).

## Results

Baseline sample characteristics

A total of 104 patients were distributed equally into four groups according to growth pattern and gender. The groups with the highest and lowest levels of attractiveness comprised 26 males and 26 females (13 per group). In the vertical growth pattern, male patients with the most and the least attractive faces had a mean age of 22.9 ± 1.42 and 21.33 ± 1.07 years, respectively. For females, the most and the least attractive faces had a mean age of 19.76 ± 1.25 and 23.75 ± 0.58 years, respectively. In the horizontal growth pattern, male patients with the most and least attractive faces had a mean age of 23.5 ± 0.42 and 21.33 ± 1.07 years, respectively. For females, the most and least attractive faces had a mean age of 20.76 ± 1.25 and 22.75 ± 0.58 years, respectively.

Influence of the raters’ gender in evaluating facial esthetics according to the patient’s growth pattern

There were statistically significant differences between the genders of raters in evaluating patients with a vertical and horizontal growth pattern (p < 0.001 and p = 0.01, respectively). The average rating of female raters was greater than that of the male raters in evaluating patients with vertical growth patterns. On the contrary, the average rating of male raters was greater than that of female raters in evaluating patients with horizontal growth patterns (Table 1).

**Table 1:** Descriptive statistics of the facial beauty scores according to the growth pattern and the rater’s gender. ^†^: Employing independent-sample t-test. SD: standard deviation; Min: minimum; Max: maximum; **: there was a statistically significant difference at p < 0.01; ***: there was a statistically significant difference at p < 0.001.

Growth pattern	Rater's Gender	Mean	SD	Min	Max	P-value^†^	Significance
Vertical	Male	33.34%	11.46%	17.08%	64.38%	<0.001	***
	Female	46.43%	9.40%	30.15%	69.70%		
Horizontal	Male	44.91%	7.97%	22.50%	60.00%	0.010	**
	Female	41.82%	5.50%	27.96%	53.15%		

Influence of the raters’ gender in evaluating facial esthetics according to the patient’s gender

There were statistically significant differences between the genders of raters in evaluating female patients with a vertical and horizontal growth pattern (p < 0.001 and p = 0.009, respectively). The average rating of the female raters was greater than that of the male raters in evaluating female patients with vertical growth patterns. On the contrary, the average rating of the male raters was greater than that of the female raters in evaluating female patients with horizontal growth patterns (Table 2).

**Table 2:** Descriptive statistics of the facial beauty scores according to the growth pattern and the patient’s gender. ^†^: Employing independent-sample t-test. SD: standard deviation; Min: minimum; Max: maximum; NS: there was no statistically significant difference at p > 0.05; **: there was a statistically significant difference at p < 0.01; ***: there was a statistically significant difference at p < 0.001.

Growth pattern	Patient gender	Male raters				Female raters				P-value^†^	Significance
		Mean	SD	Min	Max	Mean	SD	Min	Max		
Vertical	Male	37.04%	12.94%	24.92%	61.67%	43.89%	11.95%	30.15%	61.67%	0.235	NS
	Female	31.48%	10.51%	17.08%	64.38%	47.71%	7.88%	32.39%	69.70%	<0.001	***
Horizontal	Male	43.14%	8.84%	22.50%	55.42%	41.48%	6.89%	27.96%	52.41%	0.364	NS
	Female	46.05%	7.40%	34.17%	60.00%	42.04%	4.61%	34.07%	53.15%	0.009	**

## Discussion

The methodology employed in this project

The evaluation was conducted on photographs in three different modes, enabling laypersons to conduct a thorough aesthetic assessment of the face as displaying these photos collectively would achieve a more general image of the patient [5]. To prevent prejudice stemming from features of the face such as color and placement of the eyebrow, skin tone, spots, and occasionally jewelry or makeup, images were transformed to black and white [23]. The images were assessed using the VAS as this technique is regarded as simple, relevant, and comprehensible and provides unbiased evaluations of the objects [24].

The panel of raters was made up of laypersons who were unrelated to the patients and were not familiar with orthodontics or aesthetics. This panel was chosen because laypersons’ opinions are considered the most objective and unbiased [25]. Furthermore, Kiekens et al. showed that seven laypersons were adequate to provide a trustworthy aesthetic assessment of photos [14]. To improve the accuracy of the findings in this study, every photograph was assessed 30 times.

Influence of the rater’s gender on evaluating facial esthetics according to the patient’s gender

The average aesthetic evaluation of the female raters was substantially greater than that of the male raters in evaluating female patients with vertical growth patterns. In contrast, the average aesthetic evaluation of the male raters was substantially greater than that of the female raters in evaluating female patients with horizontal growth patterns. Although these differences are statistically significant, their clinical significance may be a point of difference, as some practitioners may consider this percentage clinically significant, unlike others.

However, it can be said that the female raters were more lenient in evaluating female patients with vertical growth patterns. The male raters were more critical than the females; on the contrary, the male raters were more lenient in evaluating female patients with horizontal growth patterns. The female raters were more critical than the males. The reason may be attributed to the fact that the facial features of female patients with long faces are preferred by females more than males, and the facial features of female patients with short faces are preferred by males more than females.

These findings differ from those of Kiekens et al., who found no significant differences between male and female raters’ assessments. The differences can be explained by cultural factors and the difference in the geographical region of the resident committee of Belgian and Polish origin, in addition to the differences in the age of the resident committee, which ranged between 28 and 76 years [14]. Further, the study by Abu Arqoub et al. showed that there were no significant differences between the sexes in evaluating the most and least attractive male and female profiles, which indicates the existence of a similar aesthetic standard among the sexes of the evaluators. This contradiction might be due to the difference in the characteristics of the evaluating committee, such as age, professional background, and differences in the study sample, as in addition to studying vertical changes on the aesthetic evaluation of the face, the effect of sagittal changes was also studied [21]. Moreover, Samizadeh et al., who conducted an online survey, found that the gender of the evaluator did not significantly affect the selection of the two preferred options for the female profile. This can be attributed to the cultural differences of the evaluator committee, which included a general committee from society in China, as well as to the method of presenting the different patterns of the resident committee’s facial profile, which were merely demarcation shapes [26].

Regarding the results of the evaluations of vertical growth pattern patients, the results were consistent with those found in the study by Kiekens et al., where female residents noticed more aesthetic improvement in female patients after orthodontic treatment than male residents [18]. Furthermore, the Foos et al. study found that female residents rated female faces higher than males rated theirs [17]. Regarding the results of the evaluations of patients with the horizontal growth pattern, the current findings align with those of the study by Tugran et al., which found that females were more critical than males in their evaluation [27].

Study limitations

This study included Class I malocclusion patients with horizontal and vertical growth patterns and did not have different types of sagittal skeletal patterns due to several variables under evaluation. Therefore, it is necessary to conduct additional studies on the facial aesthetic evaluation of patients who have malocclusion in both the vertical and sagittal planes. Furthermore, the study’s findings are the product of laypeople’s assessment, and as different social groups may have different aesthetic standards, the outcomes of the aesthetic rating may also differ. Therefore, it can be recommended that a follow-up study be conducted to assess facial aesthetics with various panel configurations. The current sample was drawn from a single teaching hospital and was based solely on one race, limiting the generalizability of the findings. The assessment of aesthetics can vary depending on the race.

## Conclusions

Generally, the rater’s gender has a limited effect on facial esthetics. However, it can be said that the facial features of female patients with long faces are preferred by females more than males, and males are more critical in evaluating these patients. On the other hand, males favor the facial features of female patients with short faces more than females, and females are more critical in evaluating these patients. These findings suggest considering the patient’s characteristics during the diagnosis and treatment planning to obtain satisfactory results for the patient after completing orthodontic treatment. It is also preferable to know the gender of the individuals surrounding the patient and those who influence their opinion of themselves, such as the father, mother, or friend, to set a framework and ceiling for the patient’s expectations of his/her aesthetic appearance upon completion of treatment.
